# Stem cell and epithelial-mesenchymal transition markers are frequently overexpressed in circulating tumor cells of metastatic breast cancer patients

**DOI:** 10.1186/bcr2333

**Published:** 2009-07-09

**Authors:** Bahriye Aktas, Mitra Tewes, Tanja Fehm, Siegfried Hauch, Rainer Kimmig, Sabine Kasimir-Bauer

**Affiliations:** 1 Department of Gynecology and Obstetrics, University Hospital Essen, University of Duisburg-Essen, Hufelandstrasse 55, 45122 Essen, Germany; 2 Department of Internal Medicine (Cancer Research), University Hospital Essen, University of Duisburg-Essen, Hufelandstrasse 55, 45122 Essen, Germany; 3 Department of Gynecology and Obstetrics, University Hospital of Tuebingen, Calwerstrasse 7, 72076 Tuebingen, Germany; 4 AdnaGenAG, Ostpassage 7, 30853 Langenhagen, Germany

## Abstract

**Introduction:**

The persistence of circulating tumor cells (CTC) in breast cancer patients might be associated with stem cell like tumor cells which have been suggested to be the active source of metastatic spread in primary tumors. Furthermore, these cells also may undergo phenotypic changes, known as epithelial-mesenchymal transition (EMT), which allows them to travel to the site of metastasis formation without getting affected by conventional treatment. Here we evaluated 226 blood samples of 39 metastatic breast cancer patients during a follow-up of palliative chemo-, antibody – or hormonal therapy for the expression of the stem cell marker ALDH1 and markers for EMT and correlated these findings with the presence of CTC and response to therapy.

**Methods:**

2 × 5 ml blood was analyzed for CTC with the *AdnaTest BreastCancer *(AdnaGen AG) for the detection of EpCAM, MUC-1 and HER2 transcripts. The recovered c-DNA was additionally multiplex tested for three EMT markers [Twist1, Akt2, PI3Kα] and separately for the tumor stem-cell markers ALDH1. The identification of EMT markers was considered positive if at least one marker was detected in the sample.

**Results:**

97% of 30 healthy donor samples investigated were negative for EMT and 95% for ALDH1 transcripts. CTC were detected in 69/226 (31%) cancer samples. In the CTC (+) group, 62% were positive for at least one of the EMT markers and 69% for ALDH1, respectively. In the CTC (-) group the percentages were 7% and 14%, respectively. In non-responders, EMT and ALDH1 expression was found in 62% and 44% of patients, in responders the rates were 10% and 5%, respectively.

**Conclusions:**

Our data indicate that a major proportion of CTC of metastatic breast cancer patients shows EMT and tumor stem cell characteristics. Further studies are needed to prove whether these markers might serve as an indicator for therapy resistant tumor cell populations and, therefore, an inferior prognosis.

## Introduction

Recurrence in breast cancer is explained by hematogenous dissemination of tumor cells in very early stages of the disease not being detectable by common staging procedures [1,2]. Although the prognostic value of these cells has been shown by several groups [3-7], it is assumed that the metastatic potential of a tumor is based on the presence of a low number of stem cell-like tumor cells that have been identified in tumor tissue to be the active source of metastatic spread [8-10]. In this regard, one study confirmed a putative stem cell phenotype in disseminated tumor cells (DTCs) [11], and another study showed that the majority of early DTCs detected in the bone marrow of breast cancer patients with a CD44^+^/CD24^- ^phenotype correlated with a higher prevalence of bone metastases [12]. One candidate marker for a cancer stem cell phenotype is aldehyde dehydrogenase 1 (ALDH1), a detoxifying enzyme responsible for the oxidation of intracellular aldehydes [13].

However, development of metastases depends on multiple factors that determine overall tumor cell growth, survival, angiogenesis, and invasion. For epithelial malignancies, the epithelial-mesenchymal transition (EMT) is considered to be the crucial event in the metastatic process, which involves the disruption of epithelial cell homeostasis and the acquisition of a migratory mesenchymal phenotype allowing these cells to travel to the site of metastasis formation without being affected by conventional treatment [14,15]. The EMT appears to be controlled by signal-transduction pathways such as the Wnt and transforming growth factor β pathways, both of which can be aberrantly activated during neoplasia. One candidate is the TWIST gene, described to bind to E-box elements on the Akt2 promoter and to enhance its transcriptional activity and, thus, is likely to be related to the EMT phenomenon in cancer cells [16-18]. Also involved is PI3Kα, which activates the Akt1 and Akt2 Ser/Thr kinase, responsible for proliferation and antiapoptotic function [19-22].

Assuming that metastasis requires a dissemination of tumor stem cells or tumor cells showing EMT, it seems likely that such cells should be detectable among the CTCs found in the circulation of breast cancer patients.

In this study, we evaluated 226 blood samples of 39 metastatic breast cancer patients during a follow-up of palliative chemo-, antibody, or hormonal therapy for the expression of ALDH1 and the EMT markers TWIST, Akt2, and PI3Kα, and correlated these findings with the presence of CTCs and the response to therapy.

## Materials and methods

### Patient population

The study was conducted at the Department of Obstetrics and Gynecology in collaboration with the Department of Internal Medicine (Cancer Research) at the University Hospital in Essen. In total, 226 blood samples of 39 patients have been studied since October 2007.

### Eligibility criteria

The eligibility patient criteria were as follows: age, 18 years or older; measurable or evaluable metastatic breast cancer; predicted life expectancy, 2 months or more; Eastern Cooperative Oncology Group (ECOG) scores for performance status of 0 to 2; no severe uncontrolled comorbidities or medical conditions; and no second malignancies.

Patients either had a relapse of breast cancer diagnosed years before and were to start chemotherapy or had a documented progressive breast cancer before receiving a new endocrine, chemo-, or experimental therapy. Prior adjuvant treatment, radiation, or any other treatment of metastatic disease was permitted. Exclusion criteria were other malignancies except breast cancer. All specimens were obtained after written informed consent and collected by using protocols approved by the institutional review board (05/2856).

### Response criteria

Before starting a new treatment, patients underwent an evaluation of metastatic sites with ultrasound, radiographs, or computed tomography. Blood samples were collected for laboratory evaluations, including assaying CEA and Ca 15-3 plasma levels, as well as the isolation and characterization of CTCs. Reevaluations of disease status were done by the same techniques every 8 to 12 weeks, depending on the treatment schedule, until the loss or death of a patient.

Response to therapy was evaluated according to the *Response Evaluation Criteria in Solid Tumors *(RECIST): complete response (CR), disappearance of all target lesions; partial response (PR), at least 30% decrease in the sum of the LD (longest diameter) of target lesions, taking as reference the baseline sum LD; progressive disease (PD), at least 20% increase in the sum of the LD target lesions, taking as reference the smallest sum LD recorded since the treatment started or the appearance of one or more new lesions; stable disease (SD), neither sufficient shrinkage to qualify for PR nor sufficient increase to qualify for PD, taking as reference the smallest sum LD since the treatment started.

### Immunohistochemical analysis

For each of the 39 patients, the tumor type, TNM staging, and grading were assessed according to the WHO classification of tumors of the breast [23] and the sixth edition of the TNM Classification System [24] when the patients were first seen with the primary tumor. The estrogen (ER) and the progesterone (PR) receptor status were determined with immunohistochemistry. The DAKO score for the expression of HER2 was reevaluated with the HercepTest^® ^(Dako). FISH analysis in cases of 2+ staining, as determined with the HercepTest^®^, was performed as described elsewhere [25].

### IGROV cell line

The ovarian cancer cell line was purchased from the ATCC (American Tissue Culture Collection, Rockville, MD) and cultured in a humidified incubator at 37°C in an atmosphere of 5% CO_2 _and 95% air. The cell line was maintained in RPMI medium supplemented with 10% heat-inactivated fetal bovine serum and 1% penicillin/streptavidin (Biochrom KG, Seromed, Berlin, Germany).

### Sampling of biologic material

Double samples of 5 ml EDTA blood were collected for isolation of CTCs before the application of therapeutic substances with an S-Monovette^® ^(Sarstedt AG & Co, Nümbrecht, Germany) and stored at 4°C until further examination. The samples were processed immediately or not later than 4 hours after blood withdrawal. Double sampling of patient specimens was performed to avoid technical dropouts. However, the overall correspondence in two patient samples was more than 90%.

### Healthy controls

Blood (5 ml) was collected from 30 healthy donors aged 23 to 73 years for the determination of specificity and sensitivity of the applied tests for the determination of ALDH1 and EMT markers.

### Tumor cell enrichment/selection

The 226 blood samples were taken from 39 patients with metastatic breast cancer. The *AdnaTest BreastCancerSelect *(AdnaGen AG, Langenhagen, Germany) facilitates the immunomagnetic enrichment of tumor cells *via *epithelial and tumor-associated antigens. Two antibodies against the epithelial antigen MUC1 and one antibody against the epithelial glycoprotein GA733-2 (EpCAM) are conjugated to magnetic beads (Dynabeads) for the labelling of tumor cells in peripheral blood. In brief, the blood samples were incubated with a ready-to-use antibody mixture commercialized as *AdnaTest BreastCancerSelect*, according to the manufacturer's instructions. The labelled cells were extracted by a magnetic particle concentrator (MPC). Subsequently, mRNA isolation from lysed, enriched cells was performed according to the manufacturer's instructions with the Dynabeads mRNA DIRECT™ Micro Kit (Invitrogen, Karlsruhe, Germany), included in the *AdnaTest BreastCancerDetect*. Reverse transcription resulted in cDNA, which was the template for tumor cell detection and characterization by multiplex RT-PCR. Sensiscript^® ^Reverse Transcriptase (QIAGEN GmbH, Hilden, Germany) was used for the reverse transcription because of its high sensitivity (recommended for amounts of less than 50 ng RNA) in combination with oligo(dT)-coupled Dynabeads of the mRNA DIRECT™ Micro Kit, according to the manufacturer's instructions. cDNA was synthesized in a thermocycler under the following conditions: Reverse transcription was performed at 37°C for 60 minutes followed by 3 minutes at 93°C for inactivation of the reaction. The resulting cDNA was stored at -20°C until further use.

### Tumor cell detection

The *Adnatest BreastCancerDetect *was used for the detection of breast cancer-associated gene expression in immunomagnetically enriched tumor cells by reverse transcription and PCR. The analysis of tumor-associated mRNA isolated from CTC tumor cells was performed in a multiplex PCR for three tumor-associated transcripts: HER2, MUC1, and GA733-2. The thermal profile used for multiplex RT-PCR was as follows: After a 15-minute denaturation at 95°C, 35 PCR cycles were carried out by denaturation at 94°C for 1 minute, annealing/extension at 60°C for 1 minute, and elongation for 1 minute at 72°C. Subsequently, termination of the reaction was carried out at 72°C for 10 minutes, followed by storage of the samples at 4°C.

The primers generate fragments of the following sizes: GA733-2, 395 base pairs (bp); MUC1, 293 bp; and HER2, actin, 114 bp.

### The AdnaTest TumorStemCell/The AdnaTest EMT

Both tests require the enrichment of CTCs from 5 ml blood by using the *AdnaTest BreastCancerSelect *before the singleplex PCR assay to analyze ALDH1, the multiplex PCR assay to analyze EMT markers, and actin as an internal control. Contaminating leukocytes (about 1,500 per sample) are reduced 10-fold by using a special washing procedure (AdnaWash buffer). This allows the proper differentiation of EMT and tumor stem cell-expression profiles with a specificity of more than 90%, which was confirmed in healthy donor samples.

The thermal profile used for EMT multiplex RT-PCR was as follows. After a 15-min denaturation at 95°C, 35 PCR cycles were carried out by denaturation at 94°C for 30 seconds, annealing/extension at 60°C for 30 seconds, and elongation for 45 seconds at 72°C. Subsequently, termination of the reaction was carried out at 72°C for 10 minutes, followed by storage of the samples at 4°C. The primers generate fragments of the following sizes: Akt-2, 306 bp; TWIST 1, 203 bp; PI3Kα, 595 bp; and Actin, 119 bp.

The thermal profile used for ALDH1 singleplex PCR was as follows. After a 15-minute denaturation at 95°C, 35 PCR cycles were carried out by denaturation at 94°C for 30 seconds, annealing/extension at 51°C for 30 seconds, and elongation for 30 seconds at 72°C. Subsequently, termination of the reaction was carried out at 72°C for 5 minutes followed by storage of the samples at 4°C. The generated fragment size for ALDH1 is 165 bp.

### Evaluation of data

Visualization of all PCR fragments was carried out with a 2100 Bioanalyzer by using the DNA 1000 LabChips (Agilent Technologies) and the Expert Software Package (version B.02.03.SI307). The test is considered positive if a PCR fragment of at least one tumor-associated transcript is clearly detected. With the software package for evaluation of the data on the Agilent 2100 Bioanalyzer, peaks with a concentration of > 0.15 ng/μl are positive for the transcripts GA733-2, MUC1, and HER2. Peaks that are not detected at this setting are negative (concentration of less than 0.15 ng/μl). The cut-off values for the EMT markers and ALDH1 are 0.2 ng/μl for Akt2, 0.15 ng/μl for TWIST1, 0.25 ng/μl for PI3Kα, and 0.15 ng/μl for ALDH1.

## Results

### Establishment of a blood test for detection of stem cell and EMT markers

The ovarian carcinoma cell line IGROV1 was used because all markers studied were expressed by that cell line. The analytic sensitivity was determined by the detection of a low number of target cells (5 IGROV cells spiked into 5 ml blood of healthy donors) by using the *AdnaTest BreastCancer *procedure. The spiking experiments revealed 80% recovery of the IGROV cells (data not shown). Applying an amplicon cut-off value of 0.2 ng/μl for Akt2, 0.15 ng/μl for TWIST, 0.25 ng/μl for PI3Kα, and 0.15 ng/μl for ALDH1, 97% of 30 healthy donor samples investigated were negative for EMT, and 95%, for ALDH1 transcripts.

### Patients' characteristics

#### Inclusion criteria

All patients studied had metastatic breast cancer. Patients either had a relapse of breast cancer diagnosed years before and were to start chemotherapy or had a documented progressive breast cancer before receiving a new endocrine, chemo-, or experimental therapy.

Table 1 describes the patients' characteristics before onset of the study. The table is divided into three different parts: patient characteristics at the time of first diagnosis, sites of metastasis when metastasis was diagnosed, and "treatment," is divided into *Treatment at primary diagnosis *and *Treatment in the metastatic setting*.

**Table 1:** Patient characteristics before study start

Patient characteristics at first diagnosis		Number
Number of patients		39
Median age (years)		46 (range, 35–78)
Staging of the primary tumor	is	1
	I	5
	II	21
	III	6
	IV	6
		
Histology	Ductal	24
	Lobular	5
	Ductal/lobular	2
	Other*	5
	Not known	3
		
Grading	1	4
	2	16
	3	18
	Not known	1
		
Receptor expression		
		
Estrogen and progesterone receptor	Pos	26
Estrogen receptor	Pos	31
Progesterone receptor	Pos	26
HER2 overexpression	Pos (3^+^)	11
	DAKO 2^+^/FISH^-^	3
		
Sites of metastasis		
Visceral + nonvisceral		21
Visceral		11
Nonvisceral		7
Cerebral		0
		
Elevated markers	CEA	25
	Ca15-3	27
	Not known	1
		
Treatment		
		
(Neo-)adjuvant treatment at primary diagnosis		
Chemotherapy		19
Radiation		11
Hormone		18
Trastuzumab		1
Not known		0
		
Treatment in the metastatic setting		
Chemotherapy line	First	13
	Second	4
	Third	6
	Fourth and more	12
	Not known	0
Trastuzumab		11
Hormone therapy		23

*Medular, trabecular, secretory, neuroendocrine, adenocarcinoma.

In total, 39 patients were enrolled since October 2007. As shown in Table 1, the patients ranged in age from 35 to 78 years. Only 10 patients had primary metastatic breast cancer. Most patients had ductal breast cancer. Moderately and poorly differentiated tumors were predominant. Most patients had visceral and nonvisceral metastasis, and 31 and 26 primary tumors were ER and PR positive, respectively; 11 primary tumors had an overexpression of HER2 (DAKO, score 3^+^). The chemotherapeutic adjuvant treatment mostly contained anthracyclines and taxanes. Patients with metastatic tumors of the breast received different chemotherapeutic treatments including anthracylines, taxane, vinorelbine, and 5-FU. Most patients were extensively pretreated before starting the collection of the blood samples. Nineteen patients received anthracycline- or taxane-based chemotherapy in an adjuvant or metastatic setting before study start. Nearly all patients with HER2 3^+ ^tumors received trastuzumab in the metastatic setting, and only one, in the adjuvant setting.

CTC determination was performed before individual chemotherapy or hormonal treatment, depending on the pretreatment.

#### Stratifying of results

In total, 39 patients were monitored for CTCs during chemotherapy over a median period of 9 months (2 to 18 months) and stratified into responders and nonresponders. Patients (16 of 39) were monitored for between 9 and 18 months. During that period of monitoring, they were both responders and nonresponders. Thus, these patients appear twice in the calculation of results, resulting in 21 responders and 34 nonresponders for all further evaluations.

### Correlation of CTC and response

**Figure 1:**
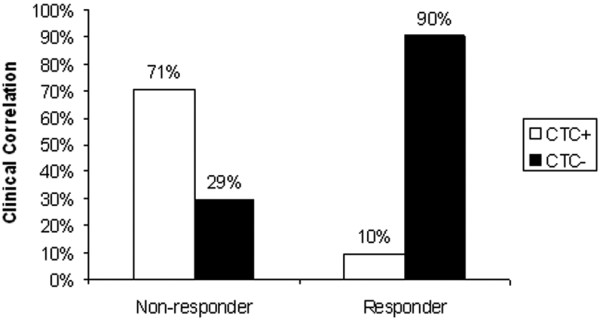
Correlation of circulating tumor cells (CTCs) and response to therapy. Patients are stratified into responders and nonresponders.

In total, CTCs were detected in 69 (31%) of all 226 cancer samples investigated during follow-up of the disease. In the CTC-positive samples investigated, at least two markers were overexpressed in the majority of the cases (60%), whereas overexpression of only one of the markers was observed in 24% of the cases for MUC1, in 13% for HER2, and 3% for Ga733-2 (data not shown). When these results were correlated with response to therapy, 24 (71%) of 34 nonresponders and 2 (10%) of 21 responders were found in the CTC^+ ^group, whereas 10 (29%) of 34 nonresponders and 19 (90%) of 21 responders were found in the CTC^- ^group (Figure 1).

### Expression of CD34

Twelve patients initially positive for CTCs and positive for at least one of the EMT markers or ALDH1 or both were further tested for CD34 to exclude potential interference of normal hematopoietic stem cells in the blood samples. However, none of the samples was positive for CD34 (data not shown).

### Correlation of CTC and EMT markers/ALDH1

**Figure 2:**
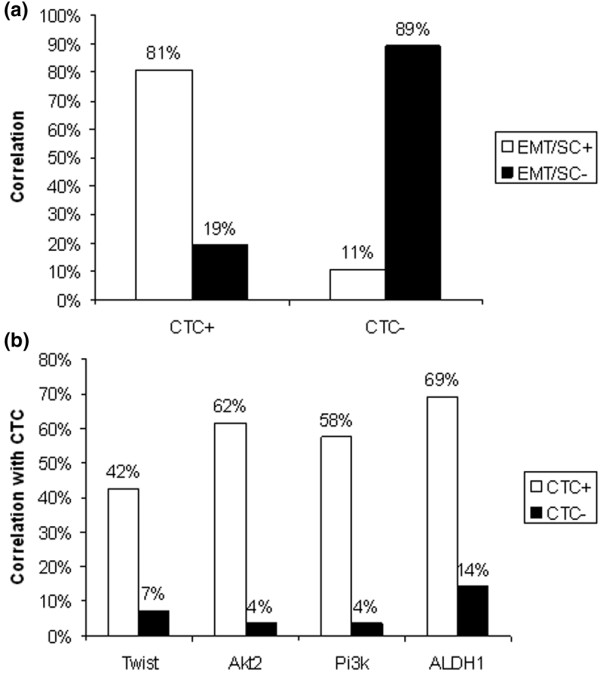
Correlation of circulating tumor cells (CTCs), epidermal/mesenchymal transition (EMT) markers, and/or ALDH1. **(a) **The identification of EMT markers was considered positive if at least one marker was detected in the sample. **(b) **Detailed analysis for the correlation of CTC and ALDH1 as well as the EMT markers.

All samples were further examined for ALDH1 and EMT markers and correlated with the presence of CTC (Figure 2a). In the CTC^+ ^group, 21 (81%) of 26 patients were positive for at least one of the EMT markers or ALDH1 or both. In the CTC^- ^group, the percentage was 11% (3 of 29) patients, respectively. Figure 2b shows a more-detailed analysis of the distribution of the individual markers among the CTC^+ ^and CTC^- ^groups.

In the CTC (^+^) group, the expression rates for the EMT markers were 42% (TWIST), 62% (Akt2), 58% (PI3Kα), and 69% for ALDH1. In the CTC^- ^group, the percentages were 7%, 4%, 4%, and 14%, respectively.

### Correlation of EMT markers/ALDH1 and response to therapy

**Figure 3:**
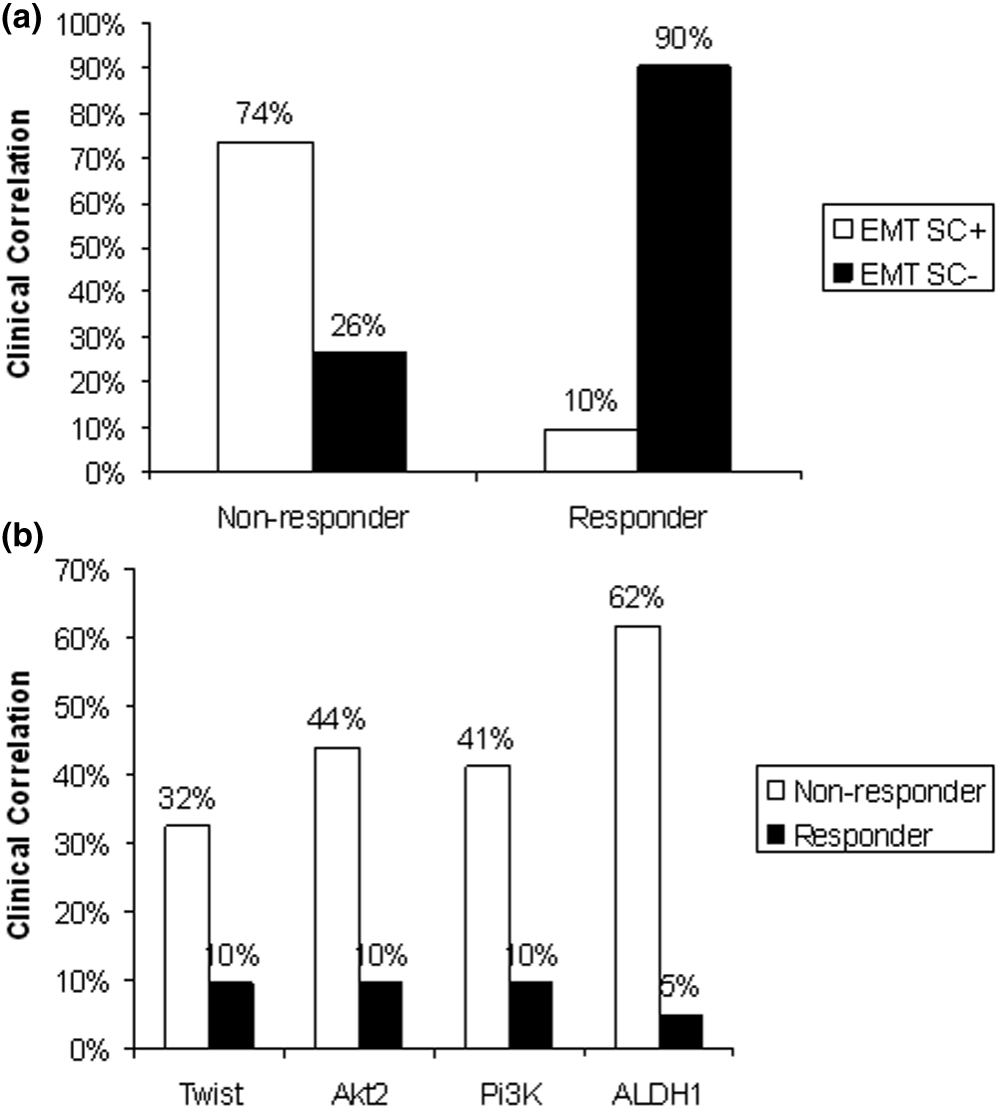
Correlation of epidermal/mesenchymal transition (EMT) markers or ALDH1 or both with response to therapy. **(a) **The identification of EMT markers was considered positive if at least one marker was detected in the sample. Patients are stratified into responders and nonresponders. **(b) **Detailed analysis for the correlation of ALDH1 as well as the EMT markers and response to therapy.

The expression of EMT or stem cell markers or both in CTCs was compared with the clinical follow-up results (Figure 3a). EMT markers or ALDH1 or both were found in 25 (74%) of 34 nonresponders and in 2 (10%) of 21 responders. In contrast, no EMT markers or ALDH1 or both were detected in 9 (26%) of 34 nonresponders and in 19 (90%) of 21 responders. Figure 3b shows a more-detailed analysis of the distribution of the individual markers among the responders and nonresponders. In the nonresponder group, the expression rate for the EMT markers were 32% (TWIST), 44% (Akt2), 41% (PI3Kα), and 62% for ALDH1. In the responder group, the percentages were 10% for each EMT marker and 5% for ALDH1.

## Discussion

Recent key findings in primary tumor tissue suggest that the metastatic potential of a tumor is based on the presence of a low number of stem cell-like tumor cells that have been identified in tumor tissue to be the active source of metastatic spread. Furthermore, it was shown that tumor cells that are spreading into the circulation may undergo phenotypic changes, known as EMT, which allows them to travel to the site of metastasis formation without being affected by conventional treatment.

Here we demonstrated that at least one or more of the EMT markers TWIST, Akt2, and PI3Kα, as well as the stem cell marker ALDH1 were detectable, mainly in patient samples that also were positive for CTCs. These findings correlated with response to breast cancer-related therapies and indicate that a major proportion of CTCs found in the blood of cancer patients shows EMT and tumor stem cell characteristics, and that CTCs expressing EMT and tumor stem cell markers are an indication for therapy-resistant cell populations and thus for an inferior prognosis.

The potential existence of a stem cell-like cell in breast cancer was shown by Al-Hajj and colleagues [26], who identified a putative breast tumor stem cell-like population that is defined by the presence of CD44 and absence of CD24. One can assume that such tumor stem cells are disseminated from the primary tumor into the circulation and escape therapy because of their stem cell properties until they reach their homing organ, where they act as seed for metastasis formation [1]. This assumption is supported by the observation that primary tumor stem cells show an expression profile associated with metastatic relapse in breast cancer patients [27]. More recently, ALDH1 was found to be a specific marker for breast cancer stem cells [28]. In our samples, ALDH1 overexpression was found in approximately 70% of the blood samples also positive for CTCs.

Certain evidence suggests that CTCs might be identified partly as cancer stem cells because of similarities such as increased resistance to chemotherapy and decreased proliferation during circulation. Similar findings were reported for DTCs in bone marrow, where tumor cells with a stem cell-like phenotype were demonstrated [11]. Corresponding experimental results for CTCs are still outstanding. Preliminary data, presented at the annual meeting of the American Association for Cancer Research 2008, identified a breast cancer stem cell-like phenotype in blood samples of patients with breast cancer [29]. We recently demonstrated that the presence or disappearance of CTCs during the time course of individual treatment is a predictor of therapy response in metastatic breast cancer [30]. In a follow-up of these patients, we now demonstrate that the expression of the stem cell marker ALDH1 among CTCs is associated with therapy failure. Assuming that breast cancer cells are very heterogeneic, the fraction of CTCs, theoretically, should include the fraction of tumor cells able to form metastasis.

Because the morphology of the cells cannot be shown with this method, we cannot exclude false-positive results in some cases. One of the molecular markers, such as MUC1 or HER2, might be overexpressed in activated leukocytes present only in cancer patient blood. However, in the major proportion of the CTC-positive samples investigated in this study, at least two markers were overexpressed in the majority of the cases (60%), whereas overexpression of only one of the markers was observed in 24% of the cases for MUC1, in 13% for HER2, and in 3% for Ga733-2. Furthermore, even if false-positive results cannot be absolutely avoided, the correlation of CTCs detected by using the AdnaTestBreastCancer as well as the EMT or stem cell-related mRNA profiles fits positively with therapy response and overall survival rate. Visualization of the cells finally will show whether the expression patterns can be confirmed to be related to CTCs only.

The two breast cancer subtypes with bad outcome are basal-like and HER2-like breast tumors. Clinically, basal-like breast cancer is characterized as the triple-negative phenotype (ER, PR, HER2, all negative) and as resembling stem-like cells, composed mainly of cells expressing the cancer stem cell markers CD44^+ ^and cytokeratin 5/6 [31-33]. In our patient group analyzed, only 1 of 39 patients was characterized as triple-negative at first diagnosis. In contrast, 11 of our patients were initially classified as HER2^+ ^on the primary tumor. Nine of these 11 patients were now among those patients not responding to different kinds of therapy. Recent work from the group of Wicha [34] suggests a strong link between HER2-like-tumors and stem cells. They reported that modulation of signaling molecules such as phosphatase and tensin homologue on chromosome 10 (PTEN) or HER2 can increase the size of the stem cell population.

The metastatic potential of a tumor depends on multiple factors that determine tumor growth, survival, angiogenesis, and invasion. In this context, EMT is considered to be one of the factors to be involved in the metastatic process. These cells have reduced apoptosis and are drug resistant. A recent report suggests that a direct link might exist between the EMT and the acquisition of stem cell properties [35]. Furthermore, cells undergoing EMT could be the precursors to metastatic cancer cells, perhaps even metastasis-forming CTCs. In our study, ALDH1 was frequently coexpressed with one of the EMT markers analyzed, resulting in cancer progression and therapy failure. Although the patient number at the moment is too small to draw any significant conclusions, during follow-up of the patients, ALDH1 and EMT markers were sometimes detected before the appearance of CTCs in the circulation. Further studies and longer follow-up times are needed to evaluate whether a systemic marker pattern can indicate early a reappearance of CTCs, and thus a progressive process of the disease.

EMT is known to occur in embryonic development, in which epithelial cells must escape structural constraints imposed by tissue architecture. They achieve this by adopting a phenotype more amenable to cell movement. The progression of carcinomas to invasive and metastatic disease shows high similarities to this process. Previous epithelial tumor cells that may convert into a mesenchymal phenotype could, therefore, escape the primary tumor tissue and develop resistance against conventional therapy regimens (for example, antihormone treatment) because they lost the relevant therapeutic targets during that transformation [36]. Conversely, it might also be possible that the expression of potential therapeutic targets, like the HER2-receptor, is induced in such cells, even if the primary tumor was found to be negative for these targets [37]. Recently published data, analyzing 16 patients with early breast cancer and 16 patients with metastatic breast cancer with immunocytochemical double staining, demonstrated that CTCs express receptors and activating signaling kinases of the EGFR/HER2/PI3K/Akt pathway, which could be used as targets for their effective elimination [38].

## Conclusions

EMT characteristics are detectable in CTCs analyzed in metastatic breast cancer samples, suggesting a negative prognostic impact of such cells because of the EMT switch that leads to decreased apoptosis and the development of chemoresistance. Furthermore, an overexpression of ALDH1, detected in a substantial number of our samples, indicates that CTCs might often display tumor stem cell characteristics, highlighting their role in metastasis formation. The detection and characterization of CTCs that show an EMT or stem cell-like metabolism could be a powerful diagnostic tool for patient stratification, the early determination of therapy failure, or the potential risk of resistance to a given therapeutic intervention. Conversely, new therapeutic strategies consisting of molecular targeting of signal-transduction pathways activated in cancer stem cells have to be developed to eliminate minimal residual disease.

## Abbreviations

ALDH1: aldehyde dehydrogenase 1; CTCs: circulating tumor cells; DTC: disseminated tumor cells; EMT: epithelial-mesenchymal transition; MRD: minimal residual disease; PD: progressive disease; PR: partial remission.

## Competing interests

The authors declare that they have no competing interests.

## Authors' contributions

BA, MT, SH, TF, and SKB made substantial contributions to the conception and design of the study, acquisition of data, and analysis and interpretation of the data. BA, TF, SH, and SKB were involved in drafting the manuscript or revising it. All authors read and approved the final manuscript.
